# A Novel ERK2 Degrader Z734 Induces Apoptosis of MCF–7 Cells via the HERC3/p53 Signaling Pathway

**DOI:** 10.3390/molecules27144337

**Published:** 2022-07-06

**Authors:** Shiyao Xu, Yan Xiong, Rui Yao, Rong Tian, Zhuqing Meng, Mohamed Y. Zaky, Beibei Fu, Dong Guo, Lulu Wang, Feng Lin, Xiaoyuan Lin, Haibo Wu

**Affiliations:** 1 School of Life Sciences, Chongqing University, Chongqing 401331, China; 202026021020@cqu.edu.cn (S.X.); 20202601005@cqu.edu.cn (Y.X.); 20192601789@cqu.edu.cn (B.F.); 20212601004@cqu.edu.cn (D.G.); 202126021005@cqu.edu.cn (L.W.); 202126021030@cqu.edu.cn (F.L.); 2 Department of Pathology, Chongqing Hygeia Hospital, Chongqing 401331, China; ruiy0102@126.com (R.Y.); tr991708389@163.com (R.T.); 3 Department of Pharmacy, Mianyang Fulin Hospital, Mianyang 621000, China; mzq15082180763@163.com; 4 Molecular Physiology Division, Zoology Department, Faculty of Science, Beni-Suef University, Beni Suef 62511, Egypt; mohamedzaki448@science.bsu.edu.eg

**Keywords:** breast cancer, ERK2 degrader, apoptosis, HERC3, p53, synergy

## Abstract

Breast cancer is one of the leading causes of death worldwide, and synthetic chemicals targeting specific proteins or various molecular pathways for tumor suppression, such as ERK inhibitors and degraders, have been intensively investigated. The targets of ERK participate in the regulation of critical cellular mechanisms and underpin the progression of anticancer therapy. In this study, we identified a novel small molecule, which we named Z734, as a new mitogen–activated protein kinase 1 (ERK2) degrader and demonstrated that Z734 inhibits cell growth by inducing p53–mediated apoptotic pathways in human breast cancer cells. Treatment with Z734 resulted in the inhibition of cancer cell proliferation, colony formation and migration invasion, as well as cancer cell death via apoptosis. In addition, the Co–IP and GST pulldown assays indicated that the HECT and RLD domains containing E3 ubiquitin protein ligase 3 (HERC3) could directly interact with ERK2 through the HECT domain, promoting ERK2 ubiquitination. We also observed a strong link between HERC3 and p53 for the modulation of apoptosis. HERC3 can increase the protein and phosphorylation levels of p53, which further promotes apoptotic activity. In a xenograft mouse model, the effect was obtained in a treatment group that combined Z734 with lapatinib compared with that of the single–treatment groups. In summary, our results indicated that Z734 actively controls the development of breast cancer through apoptosis, and HERC3 may mediate ERK2 and p53 signaling, which offers new potential targets for clinical therapy.

## 1. Introduction

Among all cancers, breast cancer has the highest incidence rate [1]. It remains a leading cause of cancer death in women worldwide. An increased understanding of the oncogenic process has driven the development of molecular target agents for cancer therapy. For the majority of breast cancer patients with inoperable tumors, targeted therapies are the best treatment option available. As signal transduction modulators, inhibitors play a critical role in cell survival or tumor progression [2]. Although targeted therapies in breast cancer have improved patient outcomes after treatment in a subset of patients, patients are confronted with the inevitable development of resistance months later. It is currently a big challenge for breast cancer targeted therapies. Hence, it is necessary to further identify underlying molecular agents to help resolve the malignant processes of breast cancer.

In this study, Z734, a hit compound, was obtained from a high–content screening for compounds that target breast cancer. We observed that Z734 could mediate cell apoptosis by regulating the ERK pathway. The mitogen–activated kinase–like protein (MAPK) pathway has been reported to relay, amplify, and integrate signals from diverse stimuli and elicit an appropriate physiological response on cellular proliferation, differentiation, development, inflammatory responses, and apoptosis in mammalian cells [3]. Currently, despite a remarkable initial response to B–Raf proto–oncogene, serine/threonine kinase (BRAF) [4,5], and mitogen–activated protein kinase kinase (MEK) [6,7] inhibitors, most cancer patients subsequently relapse due to acquired resistance. The MAP kinase pathway is linear (Ras–Raf–MEK–ERK), and resistance to the Raf and MEK inhibitors is very often the result of the reactivation of this signaling module [8]. Combined treatment with a BRAF/MEK inhibitor has been shown to block p–ERK reactivation in melanoma cells, and this may reduce the occurrence of clinical resistance [9]. The rationale for these strategies is to improve the quality and duration of clinical responses achieved by MEK inhibitors and to overcome acquired resistance, which includes ERK1/2 activation through a feedback mechanism [10,11]. This renders ERK1/2 an ideal target for overcoming resistance to both the BRAF and MEK inhibitors. In fact, modulation of ERK1/2 levels could be a particularly advantageous approach in comparison with pharmacological inhibition, since a degradation mechanism potentially induces a fast and long–lasting depletion of the target proteins, thus blocking downstream signaling cascades. To the best of our knowledge, targeted protein degradation is a new therapeutic paradigm that has been explored as a potential therapeutic strategy [12,13,14].

Approximately 80% of intracellular proteins are bound to ubiquitin, a labeling protein that sends degradation signals by recognizing the proteasome complex. By regulating the ubiquitination of proteins, the ubiquitin–proteasome system (UPS) can govern cell fate decisions, such as cell death, cell differentiation, autophagy, and senescence, and further exert essential functions in regulating homeostasis [15]. One of the ubiquitinating enzymes, HERC3, functions in the ubiquitin–proteasome machinery. The protein contains both the HECT domains necessary for the transfer of ubiquitin and the regulator of chromosome condensation (RCC1) [16,17]. Relevant research on HERC3 in terms of cancers is rare, and HERC3 was once reported to affect SMAD7 ubiquitination degradation and induce the autophagy–mediated epithelial–mesenchymal transition (EMT) and chemoresistance in glioblastomas [18]. A recent study demonstrated that HERC3 could directly interact with EIF5A2 and promote the K27– and K48–linked ubiquitination degradation of EIF5A2 to regulate EMT in colorectal cancers [15]. Through bioinformatics analysis and experimental confirmation, HERC3 was demonstrated to be downregulated in colorectal cancer tissues [15] and also to be negatively correlated with SOX18 in osteosarcomas [19].

Among the huge diversity of genes that are implicated in tumor development, the transcription factor p53, together with its complex signaling cascade, stands out as a primary regulator of cell survival or death pathways. As the “guardian of the genome”, p53 dictates cellular fates by regulating cellular responses to a plethora of environmental and intracellular stresses [20]. p53 is considered the most critical transcription factor that induces apoptosis in cancer cells [21], which is reflected by the fact that most anticancer therapeutics, including chemotherapy and radiotherapy, induce apoptosis by activating p53 [22,23]. p53 is engaged in broad crosstalk with inflammatory elements, such as MAPK pathways [24,25], and it is a multifunctional protein that plays many roles in determining a cell’s fate in response to cellular stress [26,27]. The level and function of p53 proteins are known to be regulated by posttranslational modifications, such as phosphorylation, acetylation, methylation, and ubiquitinoylation [28]. Under severe stress, p53 is activated to induce cell cycle arrest, DNA repair, senescence, or apoptosis, which have been previously demonstrated to contribute to tumor suppression [29].

In this study, a new ERK2 degrader, Z734, was found to suppress breast cancer growth by inducing cell apoptosis, and a tumor suppressor role of HERC3 in breast cancer development was revealed. We investigated the roles of ERK2 and p53 in Z734–induced apoptosis and found that Z734 regulates cell apoptosis by blocking ERK signaling and promoting HERC3/p53 signaling pathway.

## 2. Results

### 2.1. Z734 Causes Apoptosis and Inhibits MCF–7 Cells Proliferation and Migration

**Figure 1:**
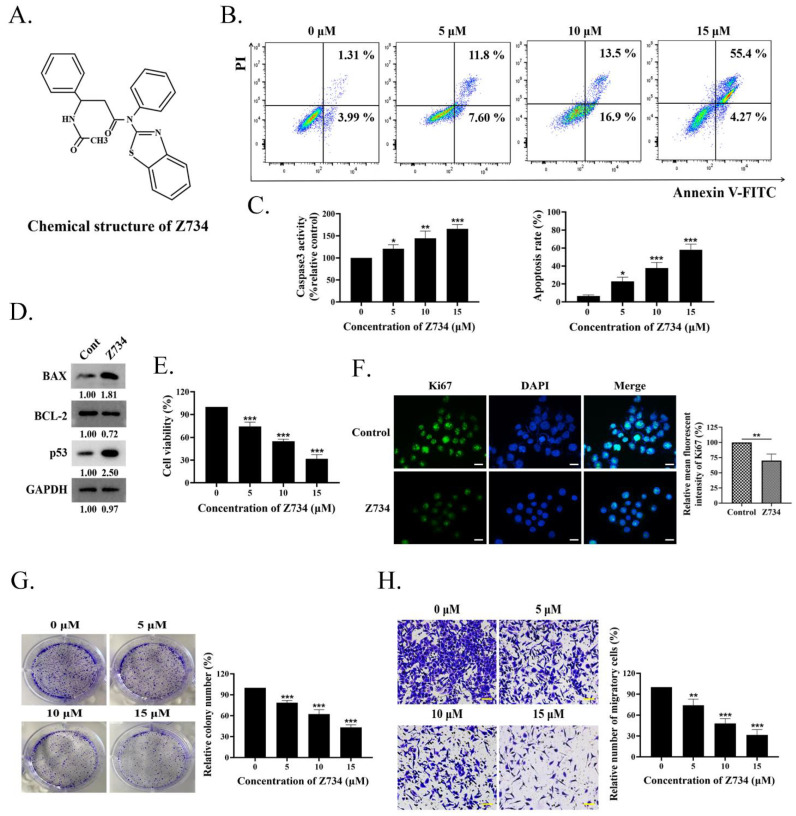
Z734 causes apoptosis and inhibits MCF–7 cells proliferation and migration. (**A**) The chemical structure of Z734. (**B**) Apoptosis levels were detected via flow cytometry. WT MCF–7 cells were treated with Z734 (0, 5, 10, and 15 μM) for 12 h. Flow cytometric analysis of apoptotic cells was conducted using AnnexinV–FITC. (**C**) Measurement of Caspase3 activity after Z734 intervention in MCF–7 cells. (**D**) Apoptosis–related protein levels after treatment with Z734 were determined via Western blot analysis. (**E**) MCF–7 cells were treated with different doses of Z734 and incubated for 12 h. Cell proliferation was assessed via CCK–8 assays. (**F**) Ki67 distribution after treatment with Z734 was determined via immunofluorescence. Quantification of fluorescence was performed on the average optical density using the ImageJ software. Scale bars = 10 μm. (**G**) The percentage of colonies of MCF–7 cell lines was determined after treatment with Z734. (**H**) MCF–7 cells were treated with Z734 for 12 h and then analyzed for migration by transwell analysis. Scale bars = 20 μm. Data shown in (**B**,**C**,**E**,**G**,**H**) were used one–way ANOVA with Tukey’s post hoc test. Data shown in (**F**) was used two–tailed Student’s *t*–test. All assays were performed in triplicate, and the data are expressed as mean ± standard deviation. * *p* < 0.05, ** *p* < 0.01, *** *p* < 0.001, n.s., no significant difference.

In a preliminary experimental design, we proposed high–content screening to identify novel potential inhibitors that target breast cancer. Based on this strategy, the compound Z734 (Figure 1A) was selected for further study. To characterize the role of this compound in the treatment of breast cancer, MCF–7 cells were treated with 0, 5, 10, and 15 μM of Z734 for 12 h. Flow cytometric analysis with propidium iodide (PI) labeling was employed to determine if Z734 affected apoptosis in MCF–7 cells. As presented in Figure 1B, the cells were mostly alive in the control group with very few dead cells, whereas treatment with Z734 significantly promoted cell apoptosis in a dose–dependent manner. The effect of Z734 on the levels of Caspase3 was further evaluated using a Caspase3 assay kit, and Caspase3 expression was observed to be enhanced after treatment with Z734 (Figure 1C). Expressions of the apoptosis–related proteins BAX and p53 were significantly increased, whereas the BCL–2 expression was reduced after treatment with Z734 (Figure 1D). CCK–8 assays revealed that Z734 significantly inhibited MCF–7 cell proliferation in a dose–dependent manner (Figure 1E). We also examined the Ki67 levels via immunofluorescence (IF), and the results indicated decreased cell proliferation after treatment with Z734 (Figure 1F). To confirm this result, a colony formation assay was performed using the MCF–7 cell line. The colony numbers significantly decreased when Z734 was used to treat MCF–7 cells (Figure 1G). Furthermore, Z734 effectively reduced the migratory capacities of MCF–7 cells, as presented in Figure 1H. Collectively, these results indicate a critical role for Z734 in MCF–7 cells by increasing cell apoptosis and inhibiting cell migration.

### 2.2. Z734 Downregulates the Expression of ERK2 in MCF–7 Cells

**Figure 2:**
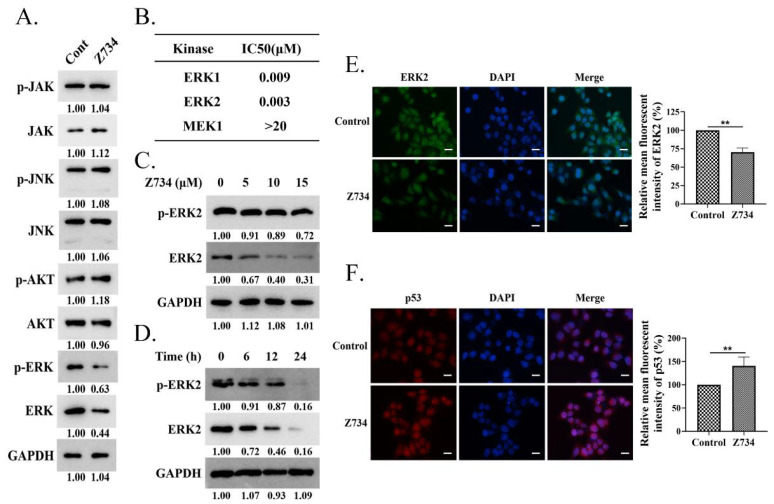
Z734 downregulates the expression of ERK2 in MCF–7 cells. (**A**) MCF–7 cells were treated with Z734 (10 μM for 12 h). The expression levels of p–JAK, JAK, p–JNK, JNK, p–AKT, AKT, p–ERK, ERK, and GAPDH were determined via Western blotting. (**B**) Effects of Z734 on kinase activity of ERK1, ERK2, and MEK1. (**C**) Western blot analysis of the p–ERK2 and ERK2 expressions in MCF–7 cells exposed to 0, 5, 10, and 15 μM of Z734 for 12 h. Relative protein expression was normalized to GAPDH. (**D**) Western blot analysis of the p–ERK2 and ERK2 expressions in MCF–7 cells treated with Z734 (10 μM) for 0, 6, 12, and 24 h. (**E**,**F**) MCF–7 cells were exposed to Z734 (10 μM) for 12 h and were then stained with antibodies for ERK2 and p53. DAPI was used to stain the nuclei. Images were obtained using a fluorescence microscope. Quantification of fluorescence was performed on the average optical density using the ImageJ software. Scale bars = 10 μm. Data shown in (**E**,**F**) were used two–tailed Student’s *t*–test. All assays were performed in triplicate, and the data are expressed as mean ± standard deviation. ** *p* < 0.01.

We next investigated the molecular mechanism by which Z734 promotes apoptosis. We measured the expression levels of JAK, JNK, AKT, ERK, and their phosphorylation levels in MCF–7 cells that were treated with Z734 to understand the pathways by which Z734 regulates apoptosis. The results indicated that the JAK, JNK, and AKT levels did not significantly change, whereas both the p–ERK and ERK levels decreased after treatment with Z734 (Figure 2A). Interestingly, it has been reported that MAPK/ERK signaling is correlated with poor survival in breast cancer patients [30]. To determine the effects of Z734 on the activity of ERK in breast cancer cells, IMAP cascade assays and Western blot analyses were conducted. ERK, as a component of the RAS–RAF–MEK–ERK cascade, is the only known substrate for the dual–specific MEK kinases and is activated by phosphorylation [31]. We measured the inhibitory concentration (IC50) value against the phosphorylation of ERK1, ERK2, and MEK1 in MCF–7 cells treated with Z734 (Figure 2B). The results indicated Z734 potently inhibited ERK1 and ERK2 activity with IC50 values of 9 and 3 nmol/L, respectively. Z734 decreased the expression of ERK2 in a concentration– and time–dependent manner (Figure 2C,D). Z734 only exhibited minimal inhibition activity against p–ERK2 in MCF–7 cells at concentrations up to 15 μM. The p–ERK2 level was quite low after 24 h Z734 treatment, which is probably due to the fact that the majority of ERK2 has been degraded. Immunofluorescence staining was performed to evaluate the expression of ERK2 in MCF–7 cells, and the results confirmed that Z734 decreased the expression of ERK2 (Figure 2E). These findings indicated that Z734 may be an ERK2 degrader. In oncogene–expressing cancer–prone cells, the Ras/Raf/MEK/ERK pathway is deregulated to facilitate tumor cell growth [31], whereas p53 induces apoptosis by target gene regulation and transcription–independent signaling [32]. Interestingly, immunofluorescence results indicated increased p53 levels after treatment with Z734 (Figure 2F).

### 2.3. ERK2 Inhibition Promotes Z734–Mediated Apoptosis in MCF–7 Cells

**Figure 3:**
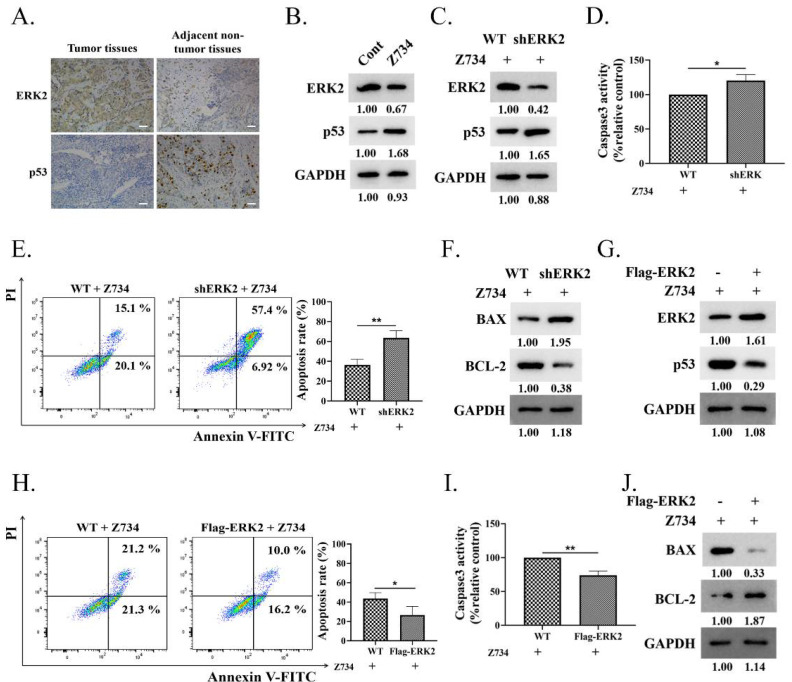
ERK2 inhibition promotes Z734–mediated apoptosis in MCF–7 cells. (**A**) ERK2 and p53 exhibit various levels of expression in tissues and in adjacent non–tumor tissues. Scale bars = 50 μm. (**B**) The ERK2 and p53 expression levels were determined via Western blotting after treatment with Z734 (10 μM). (**C**) Western blots were performed to determine the effect of ERK2 knockdown on p53 expression in MCF–7 cells after treatment with Z734 (10 μM). (**D**) Caspase3 activities in shERK2 MCF–7 cells treated with Z734 (10 μM) were determined. (**E**) WT and shERK2 MCF–7 cells were treated with Z734 (10 μM) for 12 h followed by flow cytometry to detect cell apoptosis. (**F**) Western blots were performed to detect the effect of ERK2 knockdown on BAX/BCL–2 expression in MCF–7 cells after treatment with Z734 (10 μM). (**G**) Western blots were performed to detect the effect of Flag–ERK2 on p53 expression in MCF–7 cells after treatment with Z734 (10 μM). (**H**) MCF–7 cells were transfected with Flag–ERK2 and treated with Z734 (10 μM) for 12 h followed by flow cytometry to detect cell apoptosis. (**I**) Caspase3 activities in ERK2 overexpressing MCF–7 cells treated with Z734 (10 μM) were determined. (**J**) Western blots were performed to detect the effect of overexpressing ERK2 on BAX/BCL–2 expression in MCF–7 cells after treatment with Z734 (10 μM). Data shown in (**D**,**E**,**H**,**I**) were used two–tailed Student’s *t*–test. All assays were performed in triplicate, and the data are expressed as mean ± standard deviation. * *p* < 0.05, ** *p* < 0.01.

Targeted therapies against pathways may need to consider the specific signaling context, while ERK activation plays an important role in apoptosis dependently or independently of p53 [33]. The expression levels of ERK2 and p53 in adjacent non–tumor tissues and the tumor tissues of clinical samples were determined via immunohistochemical staining. The results indicated that the protein levels of ERK2 and p53 were negatively correlated in both tumor and adjacent non–tumor tissues (Figure 3A). Then, we sought to explore the relationship between ERK2 and p53 expression in Z734–induced apoptosis in MCF–7 cells. Western blotting results indicated that treatment of cells with Z734 decreased the expression of ERK2, whereas the expression of p53 was increased (Figure 3B). After treatment with Z734, ERK2 knockdown using ERK2–specific shRNA in MCF–7 human breast cancer cells led to elevated p53 expression (Figure 3C). We further tested the effect of ERK2 on cell apoptosis after treatment with Z734 and observed that ERK2 knockdown increased the activity of Caspase3, the apoptosis rate, as well as the BAX/BCL–2 ratio, indicating an enhancement of cell apoptosis (Figure 3D–F). Overexpression of ERK2 in MCF–7 cells inhibited the expression of p53 (Figure 3G). Flow cytometry and Caspase3 assays suggested that the overexpression of ERK2 reversed the effects of Z734 on apoptosis (Figure 3H,I). In addition, the Western blot results indicated that compared with wild–type cells, the BAX/BCL–2 ratio in cells transfected with Flag–ERK2 was reduced (Figure 3J). Together, these results suggested that the down–regulation of ERK promotes p53 protein upregulation and the apoptosis of Z734–responsive.

### 2.4. HERC3 Interacts with ERK2 and Promotes in the Z734–Mediated Ubiquitination of ERK2

**Figure 4:**
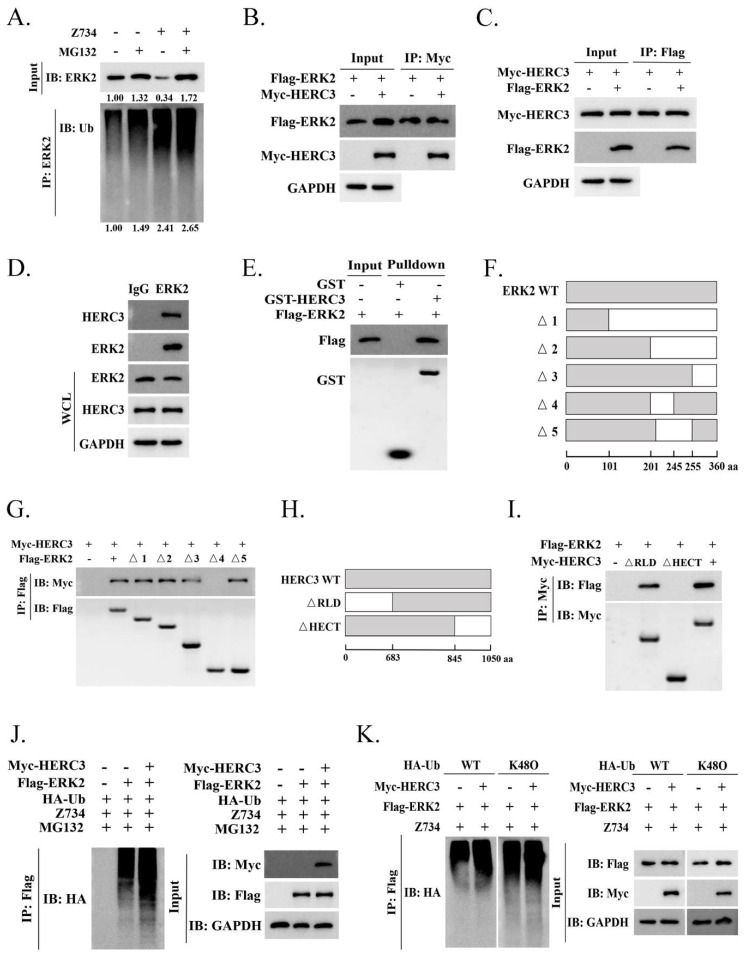
HERC3 interacts with ERK2 and promotes in the Z734–mediated ubiquitination of ERK2. (**A**) MCF–7 cells were treated with MG132 (10 mM) and Z734 (10 μM) for 12 h. (**B**) Total lysates from MCF–7 cells expressing Myc–HERC3 in the presence of Flag–ERK2 were subjected to immunoprecipitation (IP) with anti–Myc followed by Western blotting using the indicated antibodies. (**C**) Total lysates from MCF–7 cells expressing Flag–ERK2 in the presence of Myc–HERC3 were subjected to IP with anti–Flag followed by Western blotting using the indicated antibodies. (**D**) The interaction between endogenous ERK2 and HERC3 was analyzed via co–immunoprecipitation. IP samples and whole–cell lysates were analyzed via Western blotting. IgG served as the negative control. (**E**) The indicated purified GST–HERC3 protein was mixed with purified Flag–ERK2 protein. GST pulldown analyses were conducted. (**F**–**I**) Schematic representations of wild–type and deletion mutants of ERK2 (Figure 4F) and HERC3 (Figure 4H) are shown by describing each functional motif and numbers corresponding to the amino acid residues. The associations between various truncation mutants of ERK2 (Figure 4G) and HERC3 interaction–defective mutants (Figure 4I) were detected using the indicated IP and IB analyses. aa, amino acids. (**J**) MCF–7 cells were treated with MG132 (10 mM) and Z734 (10 μM) for 12 h. (**K**) MCF–7 cells were transfected with Flag–ERK2, Myc–HERC3, and wild–type (WT) or mutant ubiquitin (K48O) as indicated. The poly–ubiquitination of ERK2 was examined via Western blotting using an anti–ubiquitin antibody.

Ubiquitination regulates protein function as well as stability [34]. The ubiquitylation assays indicated that Z734 mediates ERK2 ubiquitination and proteasome blockage could alter ERK2 ubiquitination or abundance (Figure 4A). We then analyzed the potential proteins that interact with ERK2 using the web–based ubibrowser.bio (http://ubibrowser.bio-it.cn/ubibrowser/ (accessed on 11 December 2021)), and HERC3 was chosen for further study. Many components of the HERC ubiquitin protein ligases have been reported to be crucial in breast cancers. For example, HERC1, HERC4, and HERC5 have been found to be overexpressed in human breast cancer and to promote cancer progression [35,36,37,38,39]. HERC2 can physically interact with TUSC4 in breast epithelial cells and breast carcinomas to promote BRCA1 stability [40,41]. HERC3 functions as an E3 ligase in many biological processes [16,18,42], we thus wondered if ERK2 could be ubiquitinated by HERC3. To determine if HERC3 interacts with ERK2, Co–IP assays were performed. We co–transfected Flag–ERK2 and Myc–HERC3 plasmids into MCF–7 cells for 24 h. Co–IP assays confirmed that ERK2 and HERC3 can interact with each other (Figure 4B,C). Interaction between endogenous ERK2 and HERC3 was also demonstrated (Figure 4D), and the interaction was confirmed via GST pulldown assays in vitro (Figure 4E). To identify the interacting domains of ERK2 and HERC3, we generated a series of ERK2 and HERC3 deletion mutants and mapped the domains involved in binding (Figure 4F,H). Using Co–IP assays, we found that domains 201–245 of ERK2 and the HECT domain of HERC3 were necessary for their interaction (Figure 4G,I). HERC3 does involve Z734–mediated the ubiquitination of ERK2 (Figure 4J). Then, we wanted to further elucidate the mechanism by which HERC3 mediates the ubiquitination of Z734–mediated ERK2 degradation. We examined if HERC3–mediated ERK polyubiquitination is mainly through the K48–linkage. Intriguingly, ERK ubiquitination could be carried out using WT–ubiquitin (Ub) and to a more extent, K48O–Ub, indicating that ERK2 was mainly polyubiquitinated via the K48–linkage (Figure 4K). Taken together, these results suggested that HERC3 interacts with ERK2 through the HECT domain and is involved in the Z734–mediated ubiquitination of ERK2.

### 2.5. Z734–Mediated HERC3 Increasing Promotes p53 Level and p53 Phosphorylation

**Figure 5:**
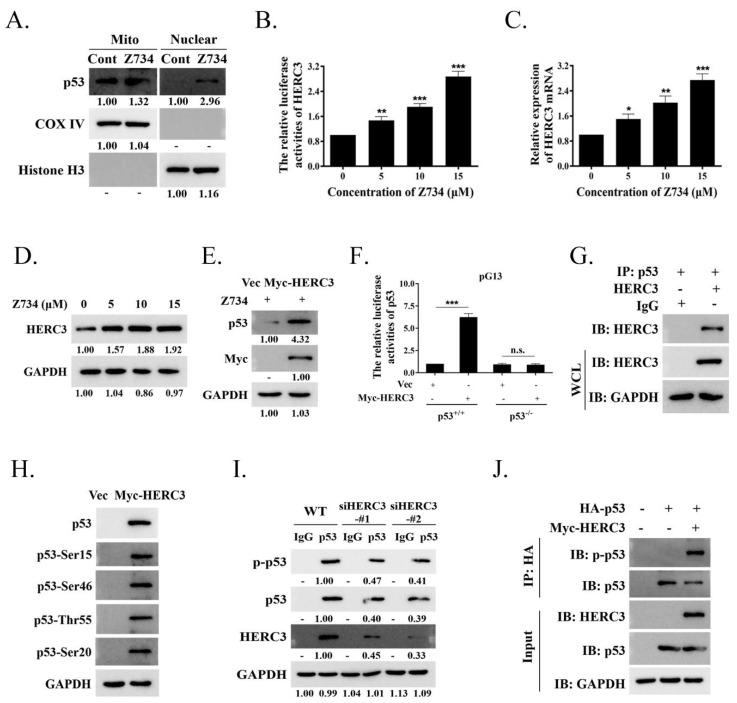
Z734–mediated HERC3 increasing promotes p53 level and p53 phosphorylation. (**A**) MCF–7 cells were treated with Z734 (10 μM) for 12 h and then mitochondrial and nuclear fractions were prepared. The levels of p53 protein in the mitochondrial, cytosolic, and nuclear fractions were assessed by Western blotting. GAPDH, cytosolic loading control; COX IV, mitochondrial loading control; Histone H3, nuclear loading control. (**B**) MCF–7 cells were transfected with a luciferase reporter containing tandem HERC3 consensus binding sites. Luciferase activity was assessed using a luciferase assay kit. (**C**) HERC3 mRNA levels in MCF–7 cells exposed to 0, 5, 10, and 15 μM Z734 for 12 h were quantified by qRT–PCR. β–actin served as the internal control. (**D**) Western blot analysis of HERC3 expression in MCF–7 cells exposed to 0, 5, 10, and 15 μM Z734 for 12 h. (**E**) MCF–7 cells were treated with Z734 (10 μM) for 12 h and transfected with Myc–HERC3, followed by Western blotting to detect p53 expression. (**F**) Wild–type (p53^+/+^) or p53 null (p53^−/−^) MCF–7 cells were transfected with empty vector or Myc–HERC3 along with a luciferase reporter containing tandem p53 consensus binding sites (pG13). Luciferase activity was assessed using a luciferase assay kit. Promoter activity is expressed as the ratio of luciferase to renilla activity (RLU, relative light units) 24 h post–transfection. (**G**) Interaction between p53 and HERC3 was analyzed by co–immunoprecipitation in MCF–7 cells. IgG served as the negative control. (**H**) Myc–HERC3 was transfected into MCF–7 cells, and expression levels of total p53 and p53 phosphorylation on Ser15, Ser46, Ser 20 or Thr55 were detected. (**I**) Knockdown of HERC3 reduces p53 level and phosphorylation. The HERC3–knockdown cells with two siRNAs targeting different sequences of HERC3 were assessed by immunoblotting. (**J**) Total lysates from MCF–7 cells expressing HA–p53 in the presence of Myc–HERC3 were subjected to IP with anti–HA, followed by Western blotting using the indicated antibodies. Data shown in (**B**,**C**) were used one–way ANOVA with Tukey’s post hoc test. Data shown in **F** was used two–tailed Student’s *t*–test. All assays were performed in triplicate, and the data are expressed as mean ± standard deviation. * *p* < 0.05, ** *p* < 0.01, *** *p* < 0.001, n.s., no significant difference.

In response to cellular stress, p53 is translocated to the nucleus to bind DNA and regulates apoptosis as a major senescence and cell death inducing transcription factor, or it is induced by the direct interactions of cytoplasmic p53 with mitochondrial proteins [29,43]. We first evaluated if Z734 also regulates the subcellular localization of p53. Mitochondrial and nuclear fractions were separated and analyzed in the presence or absence of Z734. Interestingly, we observed that p53 was present in both fractions and was enhanced after treatment with Z734 (Figure 5A). In this study, we found that the activity of the luciferase reporter driven by the HERC3 promoter (HERC3–Luc) was increased by concentration–dependent Z734 (Figure 5B). Z734 exerted an effect on the mRNA and protein levels of HERC3, and the expression was clearly increased in a concentration–dependent manner (Figure 5C,D). Next, we attempted to determine if HERC3 could regulate the p53 levels. Thus, we evaluated the possible relationship between HERC3 and p53 in the presence of Z734. We found that overexpression of HERC3 led to a higher p53 expression in MCF–7 cells after treatment with Z734 (Figure 5E). Using a luciferase reporter assay, we found that compared with the pGL3 control, the activity of the luciferase reporter driven by the HERC3 promoter (HERC3–Luc) was increased, and this effect was eliminated in p53^−/−^ MCF–7 cells (Figure 5F). Then, we attempted to determine if HERC3 interacts with p53 using Co–IP assays with MCF–7 cells, and the results confirmed a direct interaction (Figure 5G). The regulation of p53 occurs by a variety of mechanisms, including phosphorylation [44,45,46,47]. Subsequently, we investigated whether HERC3 affected p53 activity by phosphorylation. We observed that cell transfection with Myc–HERC3 can cause widespread phosphorylation in the p53 protein, including on serines 15, 46, and 20 or on threonine 55 (Figure 5H). Contrarily, HERC3 knockdown diminished the interaction of p53 with endogenous HERC3, reduced the basal levels of p53, and inhibited the phosphorylation of p53 in unstimulated MCF–7 cells (Figure 5I). To determine if p53 phosphorylation is mediated by the activity of HERC3, MCF–7 cells were transfected with HA–p53 and Myc–HERC3. As presented in Figure 5J, the levels of p–p53 were increased by overexpression of HERC3 in MCF–7 cells. Collectively, these results provide convincing evidence that HERC3 is involved in the phosphorylation at p53, thereby increasing cell apoptosis.

### 2.6. A Combination of Z734 and Lapatinib Inhibits the Growth of Breast Cancer

**Figure 6:**
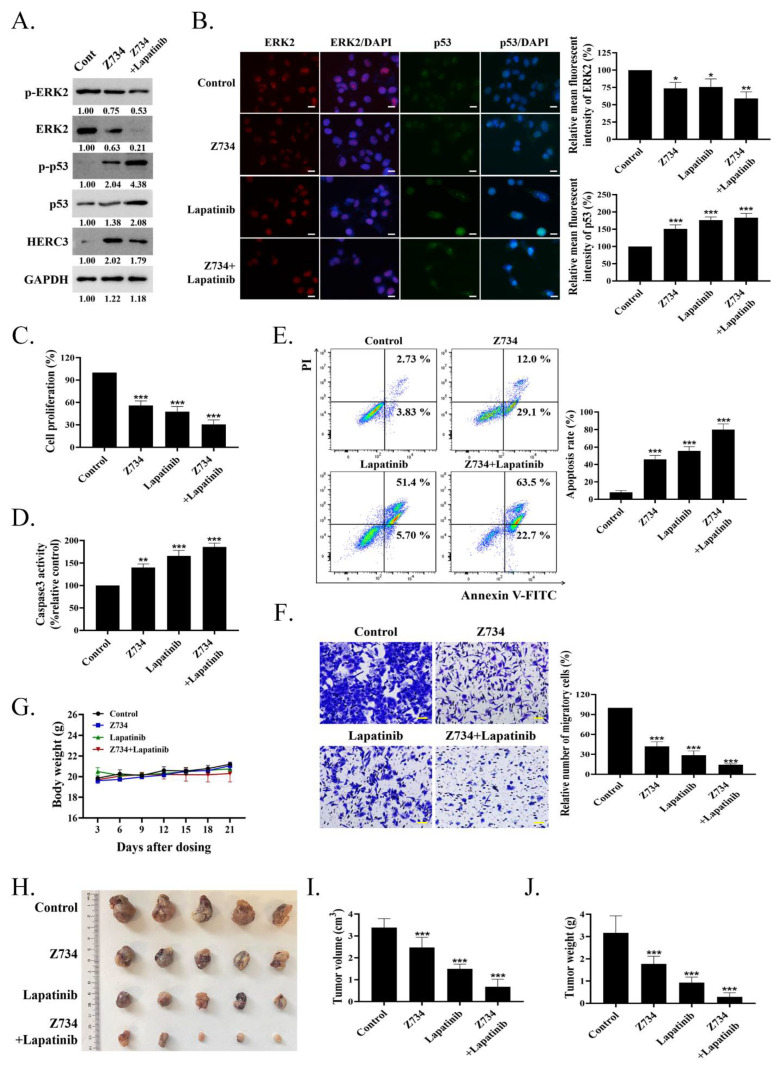
A combination of Z734 and lapatinib inhibits the growth of breast cancer. (**A**) MCF–7 cells were incubated with Z734 (10 μM) and/or lapatinib (0.5 μM), and p–ERK2, ERK2, p–p53, p53 and HERC3 protein expressions were detected by WB. (**B**) Representative images of ERK2 and p53 immunofluorescence staining in MCF–7 treated with Z734 (10 μM) or lapatinib (0.5 μM). DAPI was used to stain the nuclei. Quantification of fluorescence was performed on the Average Optical Density using the ImageJ software. Scale bars = 10 μm. (**C**) ELISA was used to detect the expression levels of Ki67 in MCF–7 to reflect cell proliferation after Z734 and/or lapatinib treatment. (**D**) Caspase3 activities in overexpressing of ERK2 in MCF–7 treated with Z734 (10 μM) and/or lapatinib (0.5 μM) were detected. (**E**) MCF–7 were treated with Z734 (10 μM) and/or lapatinib (0.5 μM) for 12 h, followed by flow cytometry to detect cell apoptosis. (**F**) Migration of MCF–7 cells were evaluated using the transwell assay after being treated with Z734 (10 μM) and/or lapatinib (0.5 μM). Scale bars = 20 μm. (**G**) Body weights of female Balb/c mice with different treatment groups (*n* = 5 for Control group; *n* = 5 for Z734 group; *n* = 5 Lapatinib group; *n* = 5 for Z734 + Lapatinib group). (**H**) Picture (*n* = 5 for Control group; *n* = 5 for Z734 group; *n* = 5 Lapatinib group; *n* = 5 for Z734 + Lapatinib group, combined results of 2 independent experiments) of MCF–7 breast tumor across four groups. (**I**,**J**) The volumes (Figure 6I) and weights (Figure 6J) were measured and quantified (*n* = 5 for Control group; *n* = 5 for Z734 group; *n* = 5 Lapatinib group; *n* = 5 for Z734 + Lapatinib group). Data shown in (**B**–**G**,**I**,**J**) were used one–way ANOVA with Tukey’s post hoc test. * *p* < 0.05, ** *p* < 0.01, *** *p* < 0.001.

Lapatinib is an oral dual tyrosine kinase inhibitor that selectively inhibits epidermal growth factor receptor/human epidermal growth factor receptor–2 (EGFR/HER2) [48]. In this study, the cells were treated with Z734 and lapatinib to determine if they could exert synergistic effects on breast cancer cells. Cotreatment with lapatinib can further repress both the ERK2 and p–ERK2 levels when compared with the control or Z734 groups (Figure 6A). The combination of Z734 and lapatinib also significantly increased the p53 and p–p53 levels. Although the addition of lapatinib did not enhance the expression of HERC3 compared with Z734 alone, the results indicated that lapatinib potentiated the action of Z734, resulting in enhanced cytotoxic efficacy. We conducted immunofluorescence analyses and ELISAs to further study the effects of Z734 and lapatinib. Figure 6B presents the expression levels of ERK2 and p53 in Z734–treated MCF–7 cells via fluorescence microscopy, and these results are consistent with the Western blot data presented in Figure 6A. We observed a decrease in cell viability with combination therapy (Z734 preincubation with lapatinib at a concentration of 10 µM) compared with cells treated with Z734 or lapatinib alone (Figure 6C). Caspase3 activities reflected increased cell apoptosis in the combination therapy treatment (Figure 6D). In addition, the cells were treated for 12 h with the agents, and cell death was analyzed via annexin V–FITC/PI staining. The treatment with Z734 increased the percentage of apoptotic cells, and a combination treatment with lapatinib further increased the apoptotic population of cells (Figure 6E). A similar synergistic effect of treatment with lapatinib and Z734 was observed for cell migration (Figure 6F). To determine if inhibition of ERK2 by Z734 is potentially clinically relevant in breast cancer, we next used breast cancer xenograft models to evaluate the effects of Z734 in vivo. We monitor animal weight as an indication of toxicity presented and no drug–related lethality was observed at all doses (Figure 6G). The subcutaneous xenograft model experiments demonstrated that tumors formed by control cells grew larger than those formed by cells treated with Z734 (Figure 6H,I). Tumor growth was significantly delayed in the combination therapy group as assessed by tumor weights after the mice were sacrificed (Figure 6J).

## 3. Discussion

To find out underlying molecular agents for breast cancer, we explored the anticancer effect of the compound Z734 and also investigated the underlying molecular mechanism. Here in this study, we explored the molecular mechanism of compound Z734 inducing apoptosis in MCF–7. We first found that Z734 promoted apoptosis and modulated cell proliferation and migration in MCF–7 cells (Figure 1). After checking for the possible involvement of different pathways, ERK2 was selected for further study as treatment with Z734 alone represses both the ERK2 and p–ERK2 levels in cell lines, whereas the expressions of AKT, JAK, and JNK were not significantly changed (Figure 2A). The MAPK pathway is involved in different cellular processes, and ERK can have different effects on the apoptotic pathway induction of drugs in cells of various lineages [33]. Our data suggested that ERK2 inhibition could increase apoptosis in MCF–7 cells under the Z734 treatment (Figure 3).

To further study the mechanism by which Z734 regulates the expression of ERK2 in tumor cells, we investigated proteins that could interact with ERK2. HERC1 had been reported to regulate cell proliferation and ERK signaling by regulating C–RAF protein levels via ubiquitylation [49], and HERC mutations and expression are associated with cancer [50,51,52,53]. In addition, it has been reported that HERC3 has ubiquitin ligase activity and is involved in the tumorigenesis of breast carcinoma [42]. Inspired by the aforementioned studies, we investigated whether ERK2 degradation was related to HERC3. Our data demonstrated that HERC3 is a ubiquitin ligase for ERK2 and can interact with ERK2 directly (Figure 4). HERC3 is involved in Z734–mediated the ubiquitination of ERK2. But we did not fully define the mechanisms by which HERC3 governs the ubiquitination of ERK without Z734, we do not rule out the possibility that other ubiquitin ligases participate in the Z734–induced downregulation of ERK2.

In our research, we also found that Z734 increased the HERC3 and p53 levels. Many studies have demonstrated that p53 can regulate apoptosis, trigger the differentiation process, and block the shift from regeneration to carcinogenesis [32,54,55]. HERC3 is highly homologous to HERC4, and it is related to p53 [56,57]. Here we demonstrated that HERC3 interacts with p53. In cancer cells, the function of p53 can be compromised [58,59]; thus, it is no longer able to suppress cell growth and proliferation as it normally does, and this contributes to the neoplastic process. The reactivation of p53 in tumors may induce cell cycle arrest and trigger apoptosis in some tumor types [60,61,62,63]. p53 is an important determinant of cancer cell apoptosis in tumor suppression, and understanding how to restore p53 function and control apoptosis could help devise better cancer therapeutic approaches. p53 signaling is a complex network that involves numerous posttranslational modifications of p53 [64]. Although we cannot yet suggest a detailed mechanism for how HERC3 triggers selective modification of a p53 residue, these results indicate that HERC3 may play a fundamental role in regulating p53. Further studies are required to understand the complete signaling networks by which HERC3 induces p53 expression.

We demonstrated in this study that Z734 may be a promising anticancer agent. Combination therapy with Z734 and lapatinib induced a significant decrease in tumor growth compared with lapatinib alone (Figure 6). As a specific kinase inhibitor of EGFR, lapatinib has been demonstrated to exhibit mild and slow inhibition profiles for ERK2 [65]. Interestingly, treatment with lapatinib led to the inhibition of HERC3. The better outcome that was produced by the two drugs may therefore be due to their divergent ability to inhibit ERK2. Taken together, a new ERK2 degrader, Z734, was found to promote apoptosis of breast cancer cells. We investigated the potential mechanism of Z734 cytotoxic activity against MCF–7 cells, which inhibits cell proliferation and migration of breast cancer by inducing the ubiquitination–based degradation of ERK2 and can further regulate the HERC3/p53 signal in breast cancer. Simultaneously, our current findings indicate a tumor suppressor role for HERC3 in breast cancer development. These results provide significant insights into the molecular mechanisms for apoptotic pathways in breast cancer after treatment with Z734.

## 4. Materials and Methods

### 4.1. Human Specimens

Breast tumor specimens (~1–4 cm^3^) and adjacent non–tumor tissues (~1 cm^3^) were obtained from the Chongqing University Cancer Hospital (Chongqing, China). Samples were procured and investigated with the approval of the institution’s review board prior to their collection, and all patients provided informed consent. Sample handling and processing were performed in accordance with the HTA guidelines.

### 4.2. Cell Lines and Transfection

MCF–7 cells were obtained from the American Type Culture Collection and were cultured using Dulbecco’s Modified Eagle’s Medium (Gibco; San Jose, CA, USA) supplemented with 10% fetal bovine serum. The cells were incubated at 37 °C in a 5% CO_2_ humidified incubator. Upon reaching confluency, the cells were detached using Trypsin–EDTA (Invitrogen, Thermo Fisher Scientific, Inc., Waltham, MA, USA). The MCF–7 cells were seeded in six–well plates and grown to 70% confluence. Plasmid DNA and synthetic siRNA were transfected into indicated cells using Lipofectamine 3000 Transfection Reagent (Invitrogen, Waltham, MA, USA), according to the manufacturer’s protocols. After 6 h of incubation at 37 °C, the cells were then maintained in normal culture media for 18 h.

### 4.3. Construction of Plasmids

siHERC3 was produced by GenePharma Company (Shanghai, China), and the WT ubiquitin was purchased from Addgene (Watertown, MA, USA). The K48O mutants were kindly provided by Yonghui Zheng (Michigan State University, East Lansing, MI, USA). Full–length coding sequences of ERK2, HERC3, and p53 (NCBI accession numbers: NM_002745.5, NM_014606, and NM_001142498.2, respectively) were cloned using PCR and inserted into the pCMV–C–Flag, pCMV–Myc, and pCMV–N–HA vectors. The shRNA sequences were inserted into a pENTR U6 vector (Invitrogen, Waltham, CA, USA). The shERK2 sequences for MCF–7 cells are as follows:

F:

5′–CACCGGACCTCATGGAAACAGATCTCGAAAGATCTGTTTCCATGAGGTCC–3′

R:

5′–AAAAGGACCTCATGGAAACAGATCTTTCGAGATCTGTTTCCATGAGGTCC–3′.

### 4.4. Real–Time Quantitative PCR (qRT–PCR)

Total RNA was isolated from MCF–7 cells by the TransZol reagent (BS259A, Biosharp, Wuhan, China). The purified RNA was reverse transcribed using an PrimeScript™ RT reagent Kit (RR037Q, Takara, Otsu, Japan). The expression of mRNA was quantified using an SYBR Premix ExTaq II Kit (RR820A, Takara, Otsu, Japan). qRT–PCR was performed on the Bio–Rad CFX–96 system (Bio–Rad, Hercules, CA, USA), and all results were normalized to β–actin mRNA levels. Data were analyzed using the 2^−ΔΔCt^ method. The primers for qRT–PCR were purchased from TSINGKE (Beijing, China). Primers are as follows: HERC3, F–CTCTGGCAGATCAGCATATCATT, R–CAGCTTTTGTATTACTGGGCA; β–actin, F–ATGCCCTGAGGCTCTTTTCC, R–CGGACTCATCGTACTCCTGC.

### 4.5. Generation of CRISPR–Cas9–Based Knockout Cells

p53^−/−^ cells were generated using the CRISPR–Cas9 method. Single–guide RNA (sgRNA) was subcloned into the plasmid, pSpCas9 (BB)–2A–Puro (PX459). The MCF–7 cells were transfected with a sequence–verified CRISPR plasmid and selected with puromycin (3 μg/mL) for 1 week. Single clones were selected under a microscope and confirmed by sequencing and immunoblotting. The target site for p53 is 5′–CCATTGTTCAATATCGTCCG–3′.

### 4.6. Cell Viability Assays

The Cell Counting Kit (CCK–8) (Beyotime Biotechnology, Shanghai, China) is a sensitive colorimetric assay used to determine the number of viable cells during cell proliferation. At least three parallel tests were conducted according to the following instructions: the MCF–7 cells were seeded into 96–well plates at a concentration of 5 × 10^3^ cells/well and cultured for 12 h; then, the medium was replaced with medium containing graded doses of Z734 (0, 5, 10, and 15 μM) and cultured for 12 h. The CCK–8 reagent was added to the medium at a 1:10 ratio and then incubated at 37 °C for 2 h. The absorbance was measured at 450 nm using a microplate reader.

### 4.7. Luciferase Reporter Assay

The MCF–7 cells were plated at 2 × 10^5^ cells/35 mm dish and transfected 12 h later with HERC3–Luc plasmid. At 12 h after the transfection, cells were treated with Z734 (0, 5, 10, and 15 μM) and cultured for 12 h. The luciferase assay was performed using Firefly Luciferase Reporter Gene Assay Kit (RG005, Beyotime, Shanghai, China). The HERC3–dependent activation of p53–responsive promoters was assessed via a luciferase assay using a dual–luciferase assay kit (Promega, Madison, WI, USA). The MCF–7 (wild–type p53 or p53^−/−^) cells were plated at 2 × 10^5^ cells/35 mm dish and transfected 12 h later with 1.0μg HERC3 plasmid or empty vector, 0.75–μg promoter construct (p53–responsive promoter pG13), and 0.25–μg Renilla as a control. After 24 h, the cells were harvested and lysed for the determination of luciferase activity using a luciferase assay kit (Promega, Madison, WI, USA), according to the manufacturer’s protocols. The luciferase activity was measured using Tropix TR717 luminometer (Thermo Fisher, Waltham, CA, USA).

### 4.8. Western Blot Analysis (WB)

The MCF–7 cells were lysed with RIPA buffer (Beyotime, Shanghai, China). Protein samples were subjected to 10% SDS–PAGE and then transferred to polyvinylidene fluoride (PVDF) membranes (Millipore, Bedford, MA, USA). The membranes were then blocked with 5% milk for 2 h at room temperature. The primary antibodies were diluted 1/500 and included Ki67 (AF173), p53 (AF7671), p–p53 (Ser15) (AF5893), p–p53 (Ser46) (AF5896), p–p53 (Thr55) (AF1696), BAX (AF0057), BCL–2 (AF0060), p–ERK1/2 (AM071), ERK1/2 (AF1051), ERK2 (AF1255), p–JAK (AF1486), JAK (AF1489), p–JNK (AJ516), JNK (AJ518), p–AKT (AF5734), AKT (AF0045), COX IV (AF6549), Histone H3 (AF0009), anti–GST (AF0174), anti–Flag (AF0036), anti–Myc (AF5054), anti–HA (AF0039) (Beyotime, Shanghai, China), p–p53 (Ser20) (ab157454) (Abcam, Wuhan, China), anti–ubiquitin (B0316), HERC3 (sc–100720) (Santa Cruz Biotechnology, Santa Cruz, CA, USA), and GAPDH (BL006B) (Biosharp, Wuhan, China). After washing with Tris–buffered saline, the membranes were incubated with the appropriate secondary antibody (A0208, A0216) (Beyotime, Shanghai, China) for 2 h at room temperature. Immunoblots were revealed using BeyoECL Plus (P0018S, Beyotime, Shanghai, China). Blots were visualized using a ChemiScope 6000 (CLiNX, Shanghai, China).

### 4.9. Immunoprecipitation (Co–IP)

To conduct co–immunoprecipitation studies (Co–IP), the lysate was centrifuged at 15,000× *g* for 15 min. The samples were then incubated with appropriate antibodies and protein A/G beads (Thermo Scientific, San Jose, CA, USA) overnight at 4 °C. The beads were washed according to the manufacturer’s protocols. Cell lysates were prepared in SDS–containing buffer, boiled, diluted, and immunoprecipitated using control IgG or anti–Flag antibodies using denaturing immunoprecipitation conditions. Western blot analyses were conducted as previously described.

### 4.10. Immunofluorescence (IF)

MCF–7 cells (2 × 10^5^/well) were seeded, fixed with 4% paraformaldehyde for 60 min, and then treated with 0.1% Triton X–100 for 60 min at room temperature. Blocking buffer (Beyotime, Shanghai, China) was used to block the cells for 60 min, and the cells were then washed with PBS three times. The cells were then incubated with corresponding primary antibodies at 4 °C overnight and then incubated with corresponding secondary antibodies for 60 min at room temperature. The cell nuclei were stained with DAPI (Beyotime, Shanghai, China) for 10 min. Fluorescence confocal images were obtained via confocal fluorescence microscopy (Zeiss Germany, Jena, Germany). A fluorescence microscope (Nikon, Shanghai, China) was used to observe the cells at 488 and 555 nm for green and red fluorescence, respectively. Three parallel tests were conducted. The mean of three randomly selected fields, at a magnification of 400×, was recorded for each slide. The mean optical density was determined to measure the fluorescence intensity using the ImageJ software. The average optical density (AOD) = (IOD SUM)/area.

### 4.11. Flow Cytometric Assays

The MCF–7 cells (2 × 10^6^/well) were cultured in six–well plates for 12 h, and the medium was replaced with medium containing Z734 (0, 5, 10, and 15 μM) and then cultured for 12 h. Follow–up experiments were conducted in accordance with the instructions supplied with the Annexin V–FITC and PI cell Apoptosis Assay Kits (BL110A, Biosharp, Wuhan, China) and were analyzed using a flow cytometer (FACSCalibur, BD Biosciences, San Jose, CA, USA). The percentage displayed is the proportion of early apoptotic cells (AnnexinV–FITC+/PI−) or late apoptotic/necrotic cells (AnnexinV–FITC+/PI+). The data were analyzed using the FlowJo V10 software (Becton Dickinson, Franklin Lakes, NJ, USA). Three parallel tests were conducted.

### 4.12. IMAP Enzymatic Assay

The procedure was performed in miniaturized kinase reaction volumes and involved the stepwise addition of enzymes, substrate and ATP. The total reaction volume was 20 μL, and Z734 (10 μM) was preincubated with the enzyme for 10 min before the addition of substrate and ATP. The kinase assay was conducted with active MEK1 and ERE2 (1.6 ng/mL), 400–nM FAM–Erktide and FAM–Mektide, and 20 μM ATP in kinase reaction buffer (10–mM Tris–HCl, pH 7.2, 10–mM MgCl_2_, 0.05% NaN_3_) containing 0.01% Tween–20 to help stabilize the enzyme. Kinase reactions were conducted for 2 h at room temperature in white standard 384–well plates (Corning Life Sciences Lowell, MA, USA), and then 60 μL of the detection mixture (1:600 dilution of IMAP binding reagent and 1:400 dilution of Terbium donor supplied by MDS Analytical Technologies, Sunnyvale, CA, USA) was added 0.5 h before reading the plate. Results were obtained using the Analyst 485/505/530 filter set. The energy transfer signal data were used to calculate the percent inhibition and the IC50 value.

### 4.13. Caspase3 Activity Assays

Caspase3 activity (C1116) was assayed using colorimetric Beyotime assay kits. The assay is based on spectrophotometric detection of the yellow chromophore *p*–nitroaniline (*p*NA) (with maximum absorbance at 405 nm) after cleavage from the conjugated substrate DEVDpNA. The MCF–7 cells (1 × 10^6^/plate) were seeded in six–well plates. After 12 h of culture, the cells were treated with Z734 (0, 5, 10, and 15 μM) for 12 h. The cells were collected and washed three times with PBS and then lysed with RIPA buffer. The cell lysates were clarified via centrifugation at 15,000× *g* for 15 min, and clear lysates containing 50 μg of protein were incubated with 100–μM enzyme–specific chlorogenic substrates at 37 °C for 12 h. The activity of Caspase3 due to the cleavage of the colorimetric substrate was determined by measuring the absorbance at 405 nm. Experiments were conducted in triplicate for each group.

### 4.14. ELISAs

Human Ki67 ELISA kits (ab253221) were purchased from Abcam, Wuhan, China. The MCF–7 cells were detached using Trypsin–EDTA. The pellets were solubilized at 2 × 10^7^ cell/mL in chilled 1 × Cell Extraction Buffer PTR for 20 min. After centrifugation at 15,000× *g* for 15 min at 4 °C, the supernatants were transferred into clean tubes, and the pellets were discarded. A portion (50 µL) of each sample and the antibody cocktail standard were added to the appropriate wells and maintained for 1 h at room temperature. After sealing the plate and incubating for 1 h, 100 µL of TMB Development Solution was added to each well, and the plate was incubated for 10 min in the dark. Finally, 100 µL of stop solution was added to each well and mixed well. The OD at 450 nm was measured, and each sample was assayed in triplicate.

### 4.15. Colony Formation Assays

The MCF–7 cells (1 × 10^3^/well) were cultured for 4 days in six–well plates. Then, the medium was replaced with medium containing Z734 (0, 5, 10, and 15 μM) for 12 h. The medium was changed every 3 days and continuously cultured for 15 days. The dishes were photographed to permit the counting of the colonies. Each concentration in the experiment was repeated three times.

### 4.16. Migration Analysis

The MCF–7 cells (1 × 10^5^/well) were added to cell migration chambers in 24–well plates. Medium containing Z734 at different concentrations (0, 5, 10, and 15 μM) was used. After 12 h of culturing, 4% paraformaldehyde was added to each chamber and then held for 60 min. The cells were stained with crystal violet and photographed, and the cells that crossed the membrane were quantified under a constant light microscope. Five pictures were taken for each sample. The experiment was repeated at least three times at each concentration.

### 4.17. Animal Studies

This study was conducted in strict accordance with the Guidelines for the Care and Use of Animals established by Chongqing University. The animal experimental procedures were approved by the Chongqing University Laboratory Animal Welfare and Ethics Committee. Female Balb/c mice, 6 to 8 weeks old, were purchased from the Hunan SJA Laboratory Animal Co., Ltd. (Changsha, China). The animals were randomly divided into four groups and were maintained under pathogen–free conditions in a 12:12–h light/dark cycle. 1 × 10^7^ MCF–7 cells were resuspended in 200 µL PBS and injected subcutaneously into the left axilla of nude mice. Drug therapy was started when the mean tumor size reached approximately 100 mm^3^. Lapatinib was dissolved in 5% Tween–80 and 45% saline and administered at a dose of 75 mg/kg by oral gavage once daily. Z734 (1.5 μg/mm^3^ tumor/day) dissolved in 10% DMSO was injected intratumorally. The control group was given 10% DMSO, 5% Tween–80, and 45% saline, respectively, and the treatment group was given lapatinib and Z734 alone or in combination for 21 days. Lapatinib (SC0204) was purchased from Beyotime (Shanghai, China). Z734 (CEPB9524048026) was purchased from Chemieliva Pharmaceutical Co., Ltd. (Chongqing, China) and dissolved in dimethyl sulfoxide (DMSO).

### 4.18. Histopathology

The tumors were fixed with 4% buffered formaldehyde for more than 24 h, embedded in paraffin, sectioned, and stained with H&E according to standard procedures. Photographs were obtained using a microscope (Carl Zeiss, Jena, Germany).

### 4.19. Statistical Analysis

Statistical analyses were conducted using GraphPad Prism 8.3 (GraphPad Holdings, San Diego, CA, USA). Data from all of the experiments are expressed as mean ± standard error. The statistical significance between the groups was determined using Student’s two–tailed unpaired *t*–test or a one–way ANOVA with Tukey’s post hoc test. All assays were performed in triplicate, and the data are expressed as mean ± standard deviation. * *p* < 0.05, ** *p* < 0.01, *** *p* < 0.001, n.s., no significant difference (Student’ attest).

## Data Availability

Data sharing is not applicable to this article as no datasets were generated or analyzed during the current study.

## Sample Availability

Samples of the compound Z734 are available from the authors.
